# 1-Bromo-2-(10β-dihydro­artemisin­oxy)ethane

**DOI:** 10.1107/S1600536810029090

**Published:** 2010-07-31

**Authors:** Marli C. Lombard, Manuel A. Fernandes, Jaco C. Breytenbach, David D. N’Da

**Affiliations:** a Department of Pharmaceutical Chemistry, North-West University, PO NWU 2520, Potchefstroom, South Africa; b Molecular Sciences Institute, School of Chemistry, University of the Witwatersrand, PO Wits 2050, Johannesburg, South Africa

## Abstract

The title compound, C_17_H_27_BrO_5_, DEB, is a derivative of artemisinin which is used in malara therapy. The OR-group at C12 is *cis* to the CH_3_-group at C11 and axially oriented on ring *D* which has a chair conformation. The crystal packing is stabilized by several weak inter­molecular C—H⋯O inter­actions, which combine to form a C—H—O bonded network parallel to (001).

## Related literature

For background to malaria, see: World Health Organisation (2008 ▶). For the effective of artemisinin analogs against malaria, see: Ploypradith (2004 ▶). For the crystal structure of artemisinin, see: Kuhn & Wang (2008 ▶) and of dihydro­artemisinin (DHA), see: Luo *et al.* (1984 ▶). Jasinski *et al.* (2008*a* ▶) redetermined the structure of DHA as well as characterizing the second polymer of β-arteether (Jasinski *et al.*, 2008*b* ▶). For the reaction of DEB with amines, see: Li *et al.* (2000 ▶). For the synthesis of artemisinin hybrids, see: Walsh *et al.* (2007 ▶); Basco *et al.* (2001 ▶); Grelepois *et al.* (2005 ▶); Gupta *et al.* (2002 ▶). For puckering analysis, see: Cremer & Pople (1975 ▶); Evans & Boeyens (1989 ▶).
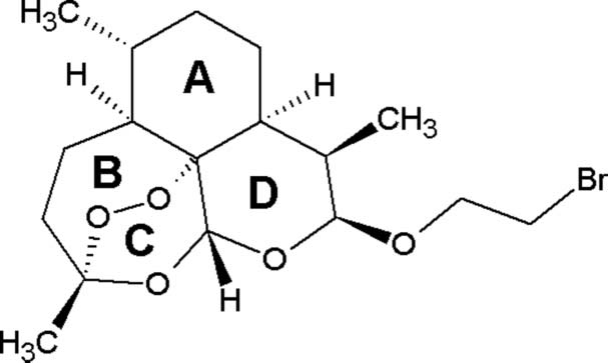


## Experimental

### 

#### Crystal data

C_17_H_27_BrO_5_
*M* *_r_* = 391.30
Monoclinic, 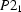

*a* = 9.2836 (2) Å
*b* = 9.1103 (2) Å
*c* = 10.2999 (2) Å
β = 90.395 (1)°
*V* = 871.11 (3) Å^3^
*Z* = 2
Mo *K*α radiation
μ = 2.38 mm^−1^
*T* = 173 K
0.44 × 0.41 × 0.08 mm

#### Data collection

Bruker APEXII CCD diffractometer
Absorption correction: integration (*XPREP*; Bruker, 2005 ▶) *T*_min_ = 0.420, *T*_max_ = 0.832
13762 measured reflections
4196 independent reflections
3432 reflections with *I* > 2σ(*I*)
*R*_int_ = 0.068

#### Refinement

*R*[*F* ^2^ > 2σ(*F* ^2^)] = 0.034
*wR*(*F* ^2^) = 0.078
*S* = 0.95
4196 reflections
211 parameters
1 restraint
H-atom parameters constrained
Δρ_max_ = 0.61 e Å^−3^
Δρ_min_ = −0.35 e Å^−3^
Absolute structure: Flack (1983 ▶), 1966 Friedel pairs
Flack parameter: −0.012 (7)

### 

Data collection: *APEX2* (Bruker, 2005 ▶); cell refinement: *SAINT* (Bruker, 2005 ▶); data reduction: *SAINT*; program(s) used to solve structure: *SHELXS97* (Sheldrick, 2008 ▶); program(s) used to refine structure: *SHELXL97* (Sheldrick, 2008 ▶); molecular graphics: *ORTEP-3 for Windows* (Farrugia, 1997 ▶) and *SCHAKAL99* (Keller, 1999 ▶); software used to prepare material for publication: *WinGX* (Farrugia, 1999 ▶) and *PLATON* (Spek, 2009 ▶).

## Supplementary Material

Crystal structure: contains datablocks global, I. DOI: 10.1107/S1600536810029090/jj2039sup1.cif

Structure factors: contains datablocks I. DOI: 10.1107/S1600536810029090/jj2039Isup2.hkl

Additional supplementary materials:  crystallographic information; 3D view; checkCIF report

## Figures and Tables

**Table 1:** Hydrogen-bond geometry (Å, °)

*D*—H⋯*A*	*D*—H	H⋯*A*	*D*⋯*A*	*D*—H⋯*A*
C15—H15*B*⋯O2^i^	0.98	2.50	3.434 (3)	159
C16—H16*A*⋯O3^ii^	0.99	2.46	3.285 (3)	141
C17—H17*B*⋯O4^ii^	0.99	2.50	3.282 (3)	136

Symmetry codes: (i) 
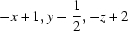
; (ii) 
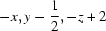
.

**Fig. 1.**
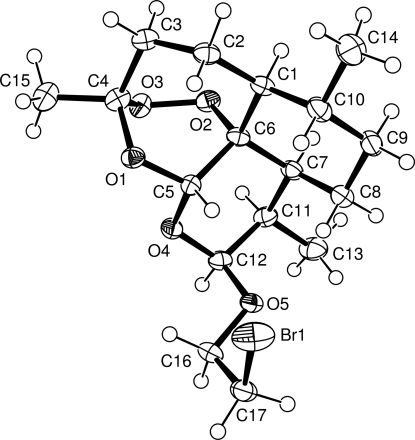
The molecular structure of C17H27BrO5 (DEB), showing the atomic numbering scheme. Displacement ellipsoids are drawn at the 50% probability level.

**Fig. 2.**
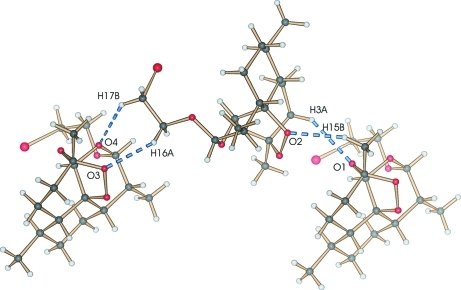
Packing diagram of C17H27BrO5 showing the weak intermolecular C—H···O hydrogen bonding network where chains of molecules run parallel to (001).

## supplementary crystallographic information

### Comment

Malaria is one of the top three priority diseases of the WHO and is becoming a worldwide threat because of its wide spread resistance to current anti-malaria drugs (World Health Organization, 2008). Artemisinin and its derivatives currently represent the most effective group of compounds against multidrug-resistant malaria with a rapid onset of action and a short half life. However, when artemisinin analogs are used as monotherapy it results in significant recrudescence (Ploypradith, 2004). Therefore it is recommended by the WHO that all uncomplicated P. falciparum infections should be treated with an artemisinin-based combination therapy (ACT).

When DHA is prepared from artemisinin by reduction, it exists as a mixture of α- and β-isomers. Luo and co-workers (Luo *et al.* 1984) determined that these isomers can exist in two conformations of ring D, a half-chair form and a half-boat form.  DEB was tested *in vitro* against *Plasmodium falciparum* sensitive (D10) and resistant (Dd2) strains, but did not show any improved activity with respect to the reference drug: dihydroartemisinin (DHA).

The title compound, C_17_H_27_BrO_5_ or 2-(10β-dihydroartemisinoxy)ethylbromide (DEB), is a derivative of artemisinin.  DEB was synthesized from the reaction of DHA with bromoethanol.  DHA was supplied as a mixture of anomers.  With the formation of DEB the OR-group at C12 is positioned *cis* to the CH_3_-group at C11 and axially oriented on ring D.  Therefore, DEB can be assigned to the β-chair series.  The rings in the title compound have also been previously labeled as rings A, B, C and D (scheme).  Ring A has a twist boat conformation with puckering parameters (Cremer & Pople, 1975) Q, θ and φ of 0.739 (2), 94.21 (15)° and 274.46 (16)°, respectively (Fig. 1).  Ring B has a distorted boat conformation and ring C is in a slightly distorted chair conformation with puckering parameters Q, θ and φ of 0.539 (3), 8.6 (3)° and 195.6 (18)°.  Ring D is in a chair conformation with puckering parameters Q, θ and φ of 0.532 (2), 178.2 (3)° and 73 (11)°.  Crystal packing in DEB is stabilized by a several C—H···O weak intermolecular interactions (Table 1) some of which are shown in Fig 2. These combine to form a C—H—O bonded network parallel to (001).

### Experimental

The title compound was prepared as described by Li and co-workers (Li *et al.*, 2000). The product was recrystallized from methanol using a slow evaporation technique at room temperature with a 71% yield of white needle-like crystals.  IC_50_ (ng/ml) of the title compound (DEB): D10 = 41.39, Dd2 = 129.47 IC_50_ (ng/ml) of Dihydroartemisinin: D10 = 1.45, Dd2 = 0.59.

### Refinement

All H atoms were positioned geometrically, and allowed to ride on their parent atoms, with Atom—H bond lengths of 1.00 Å (CH), 0.99 Å (CH_2_), or 0.98 Å (CH_3_). Isotropic displacement parameters for these atoms were set to 1.2 times (CH and CH_2_) or 1.5 times (CH_3_) *U*_eq_ of the parent atom.

### Crystal data

**Table:**

C_17_H_27_BrO_5_	*F*(000) = 408
*M**_r_* = 391.30	*D*_x_ = 1.492 Mg m^−^^3^
Monoclinic, *P*2_1_	Mo *K*α radiation, λ = 0.71073 Å
Hall symbol: P 2yb	Cell parameters from 6155 reflections
*a* = 9.2836 (2) Å	θ = 2.9–28.2°
*b* = 9.1103 (2) Å	µ = 2.38 mm^−^^1^
*c* = 10.2999 (2) Å	*T* = 173 K
β = 90.395 (1)°	Plate, colourless
*V* = 871.11 (3) Å^3^	0.44 × 0.41 × 0.08 mm
*Z* = 2	

### Data collection

**Table:**

Bruker APEXII CCD diffractometer	4196 independent reflections
Radiation source: fine-focus sealed tube	3432 reflections with *I* > 2σ(*I*)
graphite	*R*_int_ = 0.068
φ and ω scans	θ_max_ = 28.0°, θ_min_ = 2.0°
Absorption correction: integration (*XPREP*; Bruker, 2005)	*h* = −12→12
*T*_min_ = 0.420, *T*_max_ = 0.832	*k* = −12→12
13762 measured reflections	*l* = −13→13

### Refinement

**Table:**

Refinement on *F*^2^	Secondary atom site location: difference Fourier map
Least-squares matrix: full	Hydrogen site location: inferred from neighbouring sites
*R*[*F*^2^ > 2σ(*F*^2^)] = 0.034	H-atom parameters constrained
*wR*(*F*^2^) = 0.078	*w* = 1/[σ^2^(*F*_o_^2^) + (0.0405*P*)^2^] where *P* = (*F*_o_^2^ + 2*F*_c_^2^)/3
*S* = 0.95	(Δ/σ)_max_ = 0.001
4196 reflections	Δρ_max_ = 0.61 e Å^−^^3^
211 parameters	Δρ_min_ = −0.35 e Å^−^^3^
1 restraint	Absolute structure: Flack (1983), 1966 Friedel pairs
Primary atom site location: structure-invariant direct methods	Flack parameter: −0.012 (7)

### Special details

**Table:**

Experimental. ^1^H NMR (600.17 MHz; CDCl_3_; Me_4_Si): δ_H_ 5.46 (s, 1H, H-5), 4.82 (d, J = 3.4 Hz, 1H, H-12), 4.09 (ddd, J = 11.8, 6.6, 5.5 Hz, 1H, H-16α), 3.79 – 3.73 (m, 1H, H-16β), 3.51 – 3.47 (m, 2H, H-17), 2.66 – 2.59 (m, 1H, H-11), 2.39 – 2.30 (m, 1H, H-3α), 2.01 (ddd, J = 14.6, 4.7, 3.1 Hz, 1H, H-3β), 1.92 – 1.81 (m, 2H, H-2α; H-8α), 1.73 (ddd, J = 14.2, 7.7, 3.6 Hz, 1H, H-8β), 1.62 (dq, J = 13.2, 3.3 Hz, 1H, H-9β), 1.47 (qdd, J = 12.0, 8.9, 4.1 Hz, 2H, H-2α; H-7), 1.41 (s, 3H, H-15), 1.36 – 1.28 (m, 1H, H-10), 1.22 (td, J = 11.5, 6.6 Hz, 1H, H-1), 0.93 (d, J = 6.4 Hz, 3H, H-13), 0.91 (d, J = 7.4 Hz, 3H, H-14), 0.87 (dd, J = 13.3, 3.6 Hz, 3H, H-9α). ^13^C NMR (150.913 MHz; CDCl_3_; Me_4_Si): δ_C_ 104.10 (C-4), 102.02 (C-12), 88.12 (C-5), 81.07 (C-6), 68.14 (C-16), 52.54 (C-1), 44.33 (C-7), 37.36 (C-10), 36.37 (C-3), 34.63 (C-11), 31.41 (C-17), 30.86 (C-11), 26.12 (C-15), 24.62 (C-2), 24.33 (C-8), 20.34 (C-14), 12.95 (C-13).
Geometry. All e.s.d.'s (except the e.s.d. in the dihedral angle between two l.s. planes) are estimated using the full covariance matrix. The cell e.s.d.'s are taken into account individually in the estimation of e.s.d.'s in distances, angles and torsion angles; correlations between e.s.d.'s in cell parameters are only used when they are defined by crystal symmetry. An approximate (isotropic) treatment of cell e.s.d.'s is used for estimating e.s.d.'s involving l.s. planes.
Refinement. Refinement of *F*^2^ against ALL reflections. The weighted *R*-factor *wR* and goodness of fit *S* are based on *F*^2^, conventional *R*-factors *R* are based on *F*, with *F* set to zero for negative *F*^2^. The threshold expression of *F*^2^ > σ(*F*^2^) is used only for calculating *R*-factors(gt) *etc*. and is not relevant to the choice of reflections for refinement. *R*-factors based on *F*^2^ are statistically about twice as large as those based on *F*, and *R*- factors based on ALL data will be even larger.

### Fractional atomic coordinates and isotropic or equivalent isotropic displacement parameters (Å^2^)

**Table:**

	*x*	*y*	*z*	*U*_iso_*/*U*_eq_	
C1	0.4624 (2)	0.8858 (2)	0.7096 (2)	0.0219 (6)	
H1	0.4971	0.9825	0.6759	0.026*	
C2	0.5821 (3)	0.8288 (3)	0.8003 (2)	0.0240 (5)	
H2A	0.5667	0.7224	0.8142	0.029*	
H2B	0.6756	0.8404	0.7558	0.029*	
C3	0.5930 (2)	0.9028 (3)	0.9323 (2)	0.0254 (5)	
H3A	0.6188	1.0072	0.9196	0.030*	
H3B	0.6717	0.8561	0.9828	0.030*	
C4	0.4535 (2)	0.8946 (3)	1.0112 (2)	0.0236 (6)	
C5	0.2721 (2)	0.7966 (3)	0.8734 (2)	0.0193 (5)	
H5	0.2584	0.7047	0.8217	0.023*	
C6	0.3193 (2)	0.9184 (3)	0.7817 (2)	0.0199 (4)	
C7	0.1958 (2)	0.9600 (3)	0.6879 (2)	0.0221 (5)	
H7	0.2231	1.0558	0.6476	0.027*	
C8	0.1822 (3)	0.8494 (3)	0.5770 (2)	0.0293 (6)	
H8A	0.1553	0.7523	0.6126	0.035*	
H8B	0.1046	0.8812	0.5168	0.035*	
C9	0.3227 (3)	0.8356 (3)	0.5027 (2)	0.0286 (6)	
H9A	0.3101	0.7640	0.4312	0.034*	
H9B	0.3472	0.9317	0.4638	0.034*	
C10	0.4453 (3)	0.7860 (3)	0.5903 (2)	0.0268 (6)	
H10	0.4230	0.6847	0.6215	0.032*	
C11	0.0559 (3)	0.9877 (3)	0.7641 (2)	0.0246 (5)	
H11	0.0758	1.0741	0.8214	0.030*	
C12	0.0223 (3)	0.8610 (3)	0.8544 (2)	0.0230 (5)	
H12	−0.0616	0.8898	0.9092	0.028*	
C13	−0.0724 (3)	1.0314 (3)	0.6780 (3)	0.0347 (6)	
H13A	−0.1037	0.9465	0.6266	0.052*	
H13B	−0.0436	1.1110	0.6196	0.052*	
H13C	−0.1520	1.0647	0.7327	0.052*	
C14	0.5866 (3)	0.7794 (4)	0.5130 (3)	0.0378 (7)	
H14A	0.5710	0.7232	0.4331	0.057*	
H14B	0.6613	0.7317	0.5658	0.057*	
H14C	0.6173	0.8792	0.4911	0.057*	
C15	0.4802 (3)	0.8852 (3)	1.1564 (2)	0.0318 (7)	
H15A	0.5460	0.9638	1.1832	0.048*	
H15B	0.5231	0.7898	1.1776	0.048*	
H15C	0.3886	0.8959	1.2022	0.048*	
C16	−0.0657 (3)	0.6166 (3)	0.8603 (2)	0.0281 (6)	
H16A	−0.1615	0.6395	0.8966	0.034*	
H16B	0.0026	0.6017	0.9333	0.034*	
C17	−0.0743 (3)	0.4798 (3)	0.7792 (3)	0.0290 (6)	
H17A	−0.1326	0.4995	0.7003	0.035*	
H17B	−0.1227	0.4014	0.8292	0.035*	
Br1	0.11699 (3)	0.41341 (4)	0.72890 (3)	0.04547 (10)	
O1	0.37137 (18)	0.76784 (19)	0.97365 (16)	0.0221 (4)	
O2	0.34641 (18)	1.05261 (18)	0.85662 (16)	0.0241 (4)	
O3	0.3660 (2)	1.02082 (18)	0.99572 (17)	0.0234 (4)	
O4	0.13965 (17)	0.82985 (18)	0.93755 (15)	0.0225 (4)	
O5	−0.01788 (17)	0.73579 (18)	0.78053 (15)	0.0235 (4)	

### Atomic displacement parameters (Å^2^)

**Table:**

	*U*^11^	*U*^22^	*U*^33^	*U*^12^	*U*^13^	*U*^23^
C1	0.0195 (11)	0.0211 (16)	0.0252 (11)	−0.0027 (9)	0.0061 (9)	0.0001 (10)
C2	0.0186 (12)	0.0237 (12)	0.0298 (13)	0.0011 (10)	0.0059 (10)	−0.0033 (11)
C3	0.0193 (10)	0.0246 (12)	0.0322 (12)	−0.0011 (13)	0.0004 (9)	−0.0026 (14)
C4	0.0208 (11)	0.0207 (15)	0.0292 (12)	0.0039 (11)	−0.0004 (9)	−0.0050 (11)
C5	0.0199 (12)	0.0195 (12)	0.0185 (12)	−0.0010 (10)	−0.0003 (10)	0.0001 (10)
C6	0.0187 (9)	0.0162 (9)	0.0250 (11)	−0.0016 (13)	0.0032 (8)	−0.0007 (13)
C7	0.0211 (11)	0.0202 (12)	0.0250 (13)	−0.0024 (9)	0.0015 (10)	0.0066 (10)
C8	0.0266 (14)	0.0357 (13)	0.0255 (13)	−0.0069 (11)	0.0003 (11)	0.0059 (12)
C9	0.0343 (15)	0.0308 (14)	0.0206 (13)	−0.0052 (11)	0.0046 (11)	0.0010 (11)
C10	0.0322 (14)	0.0241 (12)	0.0243 (13)	−0.0034 (11)	0.0078 (11)	−0.0021 (11)
C11	0.0199 (12)	0.0233 (12)	0.0307 (14)	−0.0003 (10)	−0.0024 (11)	0.0005 (11)
C12	0.0174 (11)	0.0239 (11)	0.0277 (13)	−0.0015 (9)	0.0029 (10)	−0.0020 (10)
C13	0.0219 (13)	0.0359 (16)	0.0463 (17)	0.0016 (11)	−0.0030 (12)	0.0072 (14)
C14	0.0356 (16)	0.0446 (17)	0.0331 (15)	0.0036 (13)	0.0099 (13)	−0.0098 (13)
C15	0.0280 (12)	0.0380 (19)	0.0294 (13)	0.0083 (11)	−0.0025 (10)	−0.0077 (12)
C16	0.0265 (13)	0.0291 (14)	0.0287 (14)	−0.0081 (10)	0.0059 (11)	0.0050 (11)
C17	0.0243 (12)	0.0301 (13)	0.0326 (14)	−0.0061 (11)	0.0003 (11)	0.0075 (11)
Br1	0.03348 (15)	0.03086 (14)	0.0721 (2)	0.00014 (15)	0.00489 (12)	−0.00932 (17)
O1	0.0188 (9)	0.0213 (9)	0.0262 (10)	0.0004 (7)	−0.0008 (7)	0.0009 (8)
O2	0.0249 (9)	0.0177 (8)	0.0296 (10)	−0.0009 (7)	−0.0010 (8)	−0.0022 (7)
O3	0.0239 (9)	0.0236 (10)	0.0228 (9)	0.0051 (7)	0.0001 (7)	−0.0055 (8)
O4	0.0188 (8)	0.0270 (9)	0.0219 (8)	−0.0001 (7)	0.0026 (7)	0.0006 (7)
O5	0.0195 (8)	0.0254 (9)	0.0255 (9)	−0.0045 (7)	0.0014 (7)	0.0030 (7)

### Geometric parameters (Å, °)

**Table:**

C1—C10	1.536 (3)	C9—H9B	0.9900
C1—C2	1.538 (3)	C10—C14	1.539 (3)
C1—C6	1.556 (3)	C10—H10	1.0000
C1—H1	1.0000	C11—C12	1.515 (3)
C2—C3	1.520 (3)	C11—C13	1.533 (4)
C2—H2A	0.9900	C11—H11	1.0000
C2—H2B	0.9900	C12—O4	1.410 (3)
C3—C4	1.535 (3)	C12—O5	1.420 (3)
C3—H3A	0.9900	C12—H12	1.0000
C3—H3B	0.9900	C13—H13A	0.9800
C4—O3	1.416 (3)	C13—H13B	0.9800
C4—O1	1.435 (3)	C13—H13C	0.9800
C4—C15	1.517 (3)	C14—H14A	0.9800
C5—O1	1.404 (3)	C14—H14B	0.9800
C5—O4	1.433 (3)	C14—H14C	0.9800
C5—C6	1.523 (3)	C15—H15A	0.9800
C5—H5	1.0000	C15—H15B	0.9800
C6—O2	1.467 (3)	C15—H15C	0.9800
C6—C7	1.541 (3)	C16—O5	1.434 (3)
C7—C8	1.528 (4)	C16—C17	1.502 (4)
C7—C11	1.543 (4)	C16—H16A	0.9900
C7—H7	1.0000	C16—H16B	0.9900
C8—C9	1.522 (4)	C17—Br1	1.949 (3)
C8—H8A	0.9900	C17—H17A	0.9900
C8—H8B	0.9900	C17—H17B	0.9900
C9—C10	1.517 (4)	O2—O3	1.472 (2)
C9—H9A	0.9900		
			
C10—C1—C2	110.89 (19)	C9—C10—C1	111.9 (2)
C10—C1—C6	114.27 (19)	C9—C10—C14	110.0 (2)
C2—C1—C6	112.98 (18)	C1—C10—C14	110.7 (2)
C10—C1—H1	106.0	C9—C10—H10	108.0
C2—C1—H1	106.0	C1—C10—H10	108.0
C6—C1—H1	106.0	C14—C10—H10	108.0
C3—C2—C1	115.91 (19)	C12—C11—C13	113.0 (2)
C3—C2—H2A	108.3	C12—C11—C7	111.41 (19)
C1—C2—H2A	108.3	C13—C11—C7	113.7 (2)
C3—C2—H2B	108.3	C12—C11—H11	106.0
C1—C2—H2B	108.3	C13—C11—H11	106.0
H2A—C2—H2B	107.4	C7—C11—H11	106.0
C2—C3—C4	113.6 (2)	O4—C12—O5	111.26 (19)
C2—C3—H3A	108.8	O4—C12—C11	111.38 (19)
C4—C3—H3A	108.8	O5—C12—C11	109.76 (19)
C2—C3—H3B	108.8	O4—C12—H12	108.1
C4—C3—H3B	108.8	O5—C12—H12	108.1
H3A—C3—H3B	107.7	C11—C12—H12	108.1
O3—C4—O1	108.64 (17)	C11—C13—H13A	109.5
O3—C4—C15	104.26 (19)	C11—C13—H13B	109.5
O1—C4—C15	107.6 (2)	H13A—C13—H13B	109.5
O3—C4—C3	112.7 (2)	C11—C13—H13C	109.5
O1—C4—C3	110.2 (2)	H13A—C13—H13C	109.5
C15—C4—C3	113.08 (19)	H13B—C13—H13C	109.5
O1—C5—O4	105.13 (17)	C10—C14—H14A	109.5
O1—C5—C6	113.71 (18)	C10—C14—H14B	109.5
O4—C5—C6	112.52 (18)	H14A—C14—H14B	109.5
O1—C5—H5	108.4	C10—C14—H14C	109.5
O4—C5—H5	108.4	H14A—C14—H14C	109.5
C6—C5—H5	108.4	H14B—C14—H14C	109.5
O2—C6—C5	109.27 (16)	C4—C15—H15A	109.5
O2—C6—C7	104.4 (2)	C4—C15—H15B	109.5
C5—C6—C7	110.62 (18)	H15A—C15—H15B	109.5
O2—C6—C1	105.43 (17)	C4—C15—H15C	109.5
C5—C6—C1	114.0 (2)	H15A—C15—H15C	109.5
C7—C6—C1	112.44 (17)	H15B—C15—H15C	109.5
C8—C7—C6	111.4 (2)	O5—C16—C17	109.00 (18)
C8—C7—C11	115.0 (2)	O5—C16—H16A	109.9
C6—C7—C11	110.25 (19)	C17—C16—H16A	109.9
C8—C7—H7	106.6	O5—C16—H16B	109.9
C6—C7—H7	106.6	C17—C16—H16B	109.9
C11—C7—H7	106.6	H16A—C16—H16B	108.3
C9—C8—C7	111.3 (2)	C16—C17—Br1	111.10 (18)
C9—C8—H8A	109.4	C16—C17—H17A	109.4
C7—C8—H8A	109.4	Br1—C17—H17A	109.4
C9—C8—H8B	109.4	C16—C17—H17B	109.4
C7—C8—H8B	109.4	Br1—C17—H17B	109.4
H8A—C8—H8B	108.0	H17A—C17—H17B	108.0
C10—C9—C8	111.6 (2)	C5—O1—C4	113.13 (18)
C10—C9—H9A	109.3	C6—O2—O3	111.59 (16)
C8—C9—H9A	109.3	C4—O3—O2	109.64 (16)
C10—C9—H9B	109.3	C12—O4—C5	115.10 (17)
C8—C9—H9B	109.3	C12—O5—C16	112.52 (17)
H9A—C9—H9B	108.0		
			
C10—C1—C2—C3	−170.2 (2)	C2—C1—C10—C14	−59.8 (3)
C6—C1—C2—C3	−40.4 (3)	C6—C1—C10—C14	171.1 (2)
C1—C2—C3—C4	57.3 (3)	C8—C7—C11—C12	75.3 (3)
C2—C3—C4—O3	−94.8 (3)	C6—C7—C11—C12	−51.6 (3)
C2—C3—C4—O1	26.8 (3)	C8—C7—C11—C13	−53.7 (3)
C2—C3—C4—C15	147.3 (2)	C6—C7—C11—C13	179.4 (2)
O1—C5—C6—O2	−56.7 (2)	C13—C11—C12—O4	−176.3 (2)
O4—C5—C6—O2	62.7 (2)	C7—C11—C12—O4	54.3 (3)
O1—C5—C6—C7	−171.20 (19)	C13—C11—C12—O5	60.0 (3)
O4—C5—C6—C7	−51.8 (3)	C7—C11—C12—O5	−69.4 (2)
O1—C5—C6—C1	60.9 (2)	O5—C16—C17—Br1	68.7 (2)
O4—C5—C6—C1	−179.69 (19)	O4—C5—O1—C4	−93.8 (2)
C10—C1—C6—O2	−159.21 (19)	C6—C5—O1—C4	29.7 (3)
C2—C1—C6—O2	72.8 (2)	O3—C4—O1—C5	32.8 (2)
C10—C1—C6—C5	80.9 (2)	C15—C4—O1—C5	145.10 (19)
C2—C1—C6—C5	−47.1 (3)	C3—C4—O1—C5	−91.2 (2)
C10—C1—C6—C7	−46.0 (3)	C5—C6—O2—O3	18.1 (2)
C2—C1—C6—C7	−174.0 (2)	C7—C6—O2—O3	136.50 (16)
O2—C6—C7—C8	163.64 (17)	C1—C6—O2—O3	−104.81 (18)
C5—C6—C7—C8	−78.9 (2)	O1—C4—O3—O2	−72.2 (2)
C1—C6—C7—C8	49.8 (3)	C15—C4—O3—O2	173.27 (17)
O2—C6—C7—C11	−67.5 (2)	C3—C4—O3—O2	50.3 (2)
C5—C6—C7—C11	50.0 (3)	C6—O2—O3—C4	42.6 (2)
C1—C6—C7—C11	178.7 (2)	O5—C12—O4—C5	65.7 (2)
C6—C7—C8—C9	−56.9 (3)	C11—C12—O4—C5	−57.1 (2)
C11—C7—C8—C9	176.8 (2)	O1—C5—O4—C12	−179.24 (18)
C7—C8—C9—C10	59.5 (3)	C6—C5—O4—C12	56.5 (2)
C8—C9—C10—C1	−54.3 (3)	O4—C12—O5—C16	62.4 (2)
C8—C9—C10—C14	−177.7 (2)	C11—C12—O5—C16	−173.86 (19)
C2—C1—C10—C9	177.06 (19)	C17—C16—O5—C12	−167.5 (2)
C6—C1—C10—C9	48.0 (3)		

### Hydrogen-bond geometry (Å, °)

**Table:**

*D*—H···*A*	*D*—H	H···*A*	*D*···*A*	*D*—H···*A*
C15—H15B···O2^i^	0.98	2.50	3.434 (3)	159
C16—H16A···O3^ii^	0.99	2.46	3.285 (3)	141
C17—H17B···O4^ii^	0.99	2.50	3.282 (3)	136

Symmetry codes: (i) −*x*+1, *y*−1/2, −*z*+2; (ii) −*x*, *y*−1/2, −*z*+2.
